# Physical and surface analysis of calcium silicate Clinker after acid challenge: a micro-CT and SEM study

**DOI:** 10.1590/1807-3107bor-2026.vol40.051

**Published:** 2026-07-20

**Authors:** Karina Ines Medina Carita TAVARES, Airton Oliveira SANTOS-JUNIOR, Jáder Camilo PINTO, Fernanda Ferrari Esteves TORRES, Juliane Maria GUERREIRO-TANOMARU, Mário TANOMARU-FILHO

**Affiliations:** (a) Universidade Estadual Paulista – Unesp, School of Dentistry, Department of Restorative Dentistry, Araraquara, SP, Brazil.; (b) Centro Universitário Presidente Antônio Carlos – Unipac, Departament of Dentistry Barbacena, MG, Brazil.

**Keywords:** Dental Materials, Endodontics, Physical Phenomena, X-ray Microtomography, Microscopy, Electron, Scanning

## Abstract

The purpose of this study was to evaluate the effect of immersion in butyric acid (BA, pH 4.1) or phosphate-buffered saline (PBS, pH 7.0) on the properties of Clinker (CL) with particle sizes of 2 to 30 µm or < 2 µm associated with zirconium oxide and manipulated with distilled water (DW) or liquid with additives (LA) compared with Bio-C Repair (BCR) and Biodentine (BIO). Dentin tubes were prepared and filled with materials. After 24 hours, the specimens were immersed in BA or PBS (n = 5) for 7 and 28 days. Micro-computed tomography was used to evaluate volumetric change, porosity, and material/dentin interface. Surface analysis was performed by scanning electron microscopy (SEM). Statistical analyses included Kruskal-Wallis and Dunn, Mann-Whitney, Wilcoxon, unpaired *t*-test, paired *t*-test, and ANOVA and Tukey tests (α = 0.05). All groups exhibited volumetric changes similar to BCR and BIO (p > 0.05). BA significantly increased porosity (approximately 8%) compared with PBS (approximately 2%), except for CL 2 to 30 µm with LA (p < 0.05). After 28 days in BA, all groups showed increased porosity and gaps at the interface compared with baseline values (p < 0.05). CL 2 to 30 µm with DW showed greater porosity and interface gaps than the other groups (p < 0.05). SEM analysis revealed that all groups showed hydroxyapatite formation on the material surface in PBS, and structural loss in BA. Findings: Acidic pH damages the material/dentin interface, increases porosity, and promotes dimensional changes in calcium silicate cements. Distilled water without additives increases the Clinker’s porosity, interface gaps, and volume loss.

## Introduction

Calcium silicate-based materials are widely used due to their excellent biological and physicochemical properties.^1^ Their adequate filling capacity and dimensional stability reduce the possibility of bacterial leakage.^2,3^ Furthermore, the formation of an interfacial layer of hydroxyapatite resulting from the interaction of hydraulic cements with dentin improves the filling ability and highlights the bioactive potential of these materials.^4^

Different manufacturers produce materials based on calcium silicates. Angelus materials are produced from a powder called Clinker (CL; Angelus® Indústria de Produtos Odontológicos S/A, Londrina, Brazil) with different particle sizes (2 to 30 µm or < 2 µm). According to the manufacturer, the raw materials go through homogenization, drying, and calcination processes until the clinker granules which are composed of tricalcium silicate, dicalcium silicate, tricalcium aluminate, and calcium oxide, are obtained. Then, this material is submitted to a grinding process to define the particle size (2 to 30 µm or < 2 µm), and associated with a radiopacifier agent. Adequate radiopacity, alkalization capacity, cytocompatibility, and bioactivity were reported for Clinker associated with calcium tungstate as a radiopacifier after manipulation with distilled water or liquid with additives.^5,6^ However, there are still no studies that evaluate the volumetric change, porosity, and material/dentin interface of Clinker Angelus.

The particle size and type of liquid used influence the properties of calcium silicate-based materials.^6-11^ The presence of smaller particles in hydraulic repair cement improves the sealing capacity and reduces setting time, in addition to allowing greater flow and increasing their compressive strength.^6-8^ In contrast, increasing particle size has been proven to adversely affect the setting time, pH, and compressive strength of Mineral Trioxide Aggregate (MTA).^9^ As regards the components used to handle the material, the liquid with additives composed of water and a plasticizer improves handling and insertion of the cement.^10^ However, greater solubility, porosity, and gaps at the material/dentin interface have been reported when using a liquid with additives.^10,11^ Until now, there has been a lack of studies in the literature evaluating the impact of particle size and type of liquid on the physical properties of Clinker, factors that are crucial for establishing adequate clinical applications.

Apical periodontitis is related to the maintenance of infection.^12^ Inflammation leads to an acidic pH in periradicular tissues^13^ and infection within dentinal tubules,^12,14^ affecting the physicochemical properties of calcium silicate-based materials.^13,15-19^ Modifications in crystal structures have been reported for ProRoot MTA (Dentsply Maillefer, Ballaigues, Switzerland), Biodentine (Septodont, Saint-Maur-des-Fossés, France), CEM Cement (Bionique, Tehran, Iran), and Retro MTA (BioMTA, Seoul, Republic of Korea) after exposure to acidic pH.^16^ Furthermore, ProRoot MTA White (Dentsply Tulsa, Johnson City, USA) showed higher porosity and low microhardness values after an acid challenge.^15^ Moreover, Biodentine showed greater volume loss than ProRoot MTA White (Dentsply), BioAggregate (Innovative Bioceramix, Vancouver, Canada), and MTA (Angelus) after immersion in butyric acid.^13^ Similarly, Bio-C Repair (Angelus) demonstrated a higher volumetric loss when exposed to an acidic environment after 7 and 30 days.^17,19^ However, to the best of our knowledge, the effect of acidic pH on the physical properties of Clinker Angelus has not yet been reported in the literature.

Considering that repair cements based on calcium silicate can come into contact with an acidic environment in the presence of periradicular inflammation, the aim of this study was to evaluate the effect of acidic pH on the volumetric change, porosity, and material/dentin interface of Clinker with particle size from 2 to 30 µm or < 2 µm associated with the zirconium oxide radiopacifier and manipulated with distilled water or liquid with additives, using micro-computed tomography (micro-CT). Bio-C Repair and Biodentine were used as references for comparison. Analysis of the material surface was performed using scanning electron microscopy (SEM). The null hypotheses were: a) the immersion solution would not influence the physical properties of the materials; b) there would be no difference between the materials in relation to properties; and c) the immersion period would not influence the variables evaluated.

## Methods

The experimental groups, materials with their respective manufacturers, compositions, proportions, and immersion solutions are described in Table 1.

**Table 1:** Experimental groups, materials with their respective manufacturers, composition, proportion, and immersion solution.

Groups	Material and manufacturers	Composition	Proportion	Immersion solution
CL < 2 µm + 30% ZrO_2_ + DW	Clinker < 2 µm Angelus, Londrina, Brazil Lot: 64633	Powder: tricalcium silicate, dicalcium silicate, tricalcium aluminate, calcium oxide, and zirconium oxide Liquid: distilled water	1g: 450 μL (powder/liquid with additives)	Butyric acid
				PBS
CL < 2 µm + 30% ZrO_2_ + LA	Clinker < 2 µm Angelus, Londrina, Brazil Lot: 64633	Powder: tricalcium silicate, dicalcium silicate, tricalcium aluminate, calcium oxide, and zirconium oxide Liquid: water and plasticizer (polyvinylpyrrolidone)	1g: 450 μL (powder/liquid with additives)	Butyric acid
				PBS
CL 2 to 30 µm + 30% ZrO_2_ + DW	Clinker 2 to 30 µm Angelus, Londrina, Brazil Lot: 62965	Powder: tricalcium silicate, dicalcium silicate, tricalcium aluminate, calcium oxide, and zirconium oxide Liquid: distilled water	1g: 450 μL (powder/liquid with additives)	Butyric acid
				PBS
CL 2 to 30 µm + 30% ZrO_2_ + LA	Clinker 2 to 30 µm Angelus, Londrina, Brazil Lot: 62965	Powder: tricalcium silicate, dicalcium silicate, tricalcium aluminate, calcium oxide, and zirconium oxide Liquid: water and plasticizer (polyvinylpyrrolidone)	1g: 450 μL (powder/liquid with additives)	Butyric acid
				PBS
BCR	Bio-C Repair Angelus, Londrina, Brazil Lot: 57779 and 56446	Calcium silicates, calcium aluminate, calcium oxide, zirconium oxide, iron oxide, silicon dioxide, and dispersing agent	Ready to use	Butyric acid
				PBS
BIO	Biodentine Septodont, Saint Maur des Fossés, France Lot: B2622OA	Powder: tricalcium silicate, calcium oxide, calcium carbonate, iron oxides pigment, and zirconium oxide Liquid: calcium chloride and polycarboxylate	1 capsule: 6 drops of liquid	Butyric acid
				PBS

CL: Clinker; ZrO_2_: zirconium oxide; DW: distilled water; LA: liquid with additives; BCR: Bio-C Repair; BIO: Biodentine; PBS: phosphate-buffered saline.

### Sample size calculation

The G*Power 3.1.7 program for Windows (Heinrich-Heine-Universität Düsseldorf, Düsseldorf, Germany) was used for the sample calculation. A repeated-measures ANOVA test was used with an alpha level (α) of 0.05 and a test power (1–β) of 0.98 for all variables. A total of 5 specimens per group for all variables analyzed was indicated as the ideal size required to observe significant differences.

Volumetric change was considered the primary outcome, as the effect size (0.24) was used to determine the sample size. This value was derived from Janini et al.,^19^ who investigated volumetric change of repair materials under experimental conditions comparable with those in our study. Effect sizes for secondary outcomes, porosity (0.30) and the material/dentin interface (0.91), were obtained from Inada et al.,^11^ who used a similar sample model, evaluation methods, and assessment periods, ensuring methodological consistency and relevance for the present study.

### Selection and preparation of specimens

**Figure 1:**
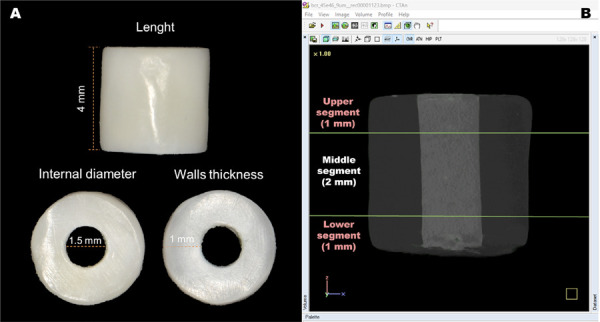
Representative image of dentin tubes. (A) parameters of bovine dentin tubes and (B) division of the sample for analysis of variables using the CTAn software.

After approval by the Ethics Committee for the Use of Animals (N° 28/2020), 60 extracted bovine teeth were radiographed (Kodak RVG 6100 Digital Radiography System, Marne-la-Vallée, France) to confirm the absence of anomalies. The middle third of each root was transversely sectioned using an Isomet 1000 cutting machine (Buehler Ltd, Lake Bluff, USA). A single previously calibrated operator prepared the root canals with a Gates-Glidden drill number 6 (Dentsply Maillefer, Ballaigues, Switzerland) coupled to the low speed motor (Micromotor N270 and Contra-angle; Dabi-Atlante, Ribeirão Preto, Brazil) and use of the delineator device (Bio-Art, São Carlos, Brazil) to ensure fixation of the samples.^18^ All dentin tubes walls were standardized to approximately 1 mm in thickness using a cylindrical drill (Maxicut 1503; American Burrs, Palhoça, Brazil), confirmed by a digital caliper measurements (Mitutoyo Corporation; São Paulo, Brazil) and digital radiography (Kodak RVG 6100 Digital Radiography System). All specimens were 4 mm long and internal diameter of 1.5 mm, as illustrated in Figure 1A. Throughout the entire preparation procedure, the canals were irrigated with a 1% sodium hypochlorite (NaOCl) solution using an Ultradent syringe (South Jordan, USA) with a 30G Navitip needle (Ultradent; South Jordan, USA). The final irrigation was performed with 5 mL of 2.5% NaOCl and 5 mL of 17% EDTA for 3 minutes, followed by 5 mL of distilled water. Subsequently, the samples were immersed in distilled water and stored in an oven at 37°C for 24 hours.^17,18^

### Filling of the specimens

After preparation, dentin tubes were randomly allocated to six groups (n = 10 per group): CL< 2 µm + ZrO_2_ + DW; CL< 2 µm + ZrO_2_ + LA; CL2 to 30 µm + ZrO_2_ + DW; CL2 to 30 µm + ZrO_2_ + LA; BCR; BIO. Each specimen was filled with its assigned cement using a condenser kit (Ref. 324501, nº 2, 3, and 4; Golgran, São Caetano do Sul, Brazil) and stored at 37°C for 24 h.

### Micro-CT scans and immersion of the specimens

The specimens were submitted to initial micro-CT scanning (SkyScan 1272, Bruker, Kontich, Belgium) with voxel size 8.74 µm, rotation step 0.5, frame 3, Aluminum filter 1 mm, rotation 180°, 80 kilovolts (kV), and 125 microamperes (uA). Within each cement group, samples were subdivided into two immersion groups (n = 5 each). A simple stratified randomization was applied based on post-obturation material volume (mm^3^), measured with CTAn v1.15.4.0 (SkyScan, Belgium). This ensured comparable baseline volumes across the immersion groups. Each specimen was individually inserted into the Eppendorf tube containing 1.5 mL of butyric acid (Sigma Aldrich, Barueri, Brazil, pH: 4.1) or phosphate-buffered saline (PBS; Sigma Aldrich, Brazil, pH: 7.0) and stored in an oven at 37 ºC for time intervals of 7 and 28 days. The butyric acid solution was replaced every 24 hours at the same time of day. Before each replacement, pH was measured with a calibrated digital pH meter (Mettler Toledo, Columbus, USA). This procedure ensured stable and controlled pH conditions throughout the experiment.^17,18^Micro-CT scans after 7 and 28 days of sample immersion were performed using the same parameters described previously.

### Blinding and examiner qualifications

To minimize bias, image examiners were blinded to group allocation and to all phases preceding image analysis (specimen preparation, obturation, specimen immersion, and scanning). Quantitative micro-CT analysis was performed by an endodontic specialist (K.I.M.C.T.) with over seven years of experience in micro-CT data evaluation. Descriptive SEM analysis was performed by J.M.G.T., an expert with over twenty years of experience in SEM image interpretation.

### Micro-CT analysis

Image reconstructions in the different experimental time intervals were performed using NRecon software v.1.6.3 (SkyScan, Belgium) after defining individual parameters for each material. The 3D reconstructed images were recorded at different periods using the Data Viewer software v.1.5.1 (SkyScan, Belgium). Quantitative analyses before and after immersion in different solutions were carried out using the CTAn software (SkyScan, Belgium). Representative images were created using CTVox software v.3.2 (SkyScan, Belgium). All dentin tubules were divided into three segments: 1 mm for the upper segment, 1 mm for the lower segment, and 2 mm for the middle segment, as illustrated in Figure 1B.

The analysis of each sample began with defining the bottom and top segments. Subsequently, the volume of interest (VOI) was selected, excluding dentin. Sample binarization was performed using a density histogram with an adaptive threshold to recognize each object of interest. The total volume of the repair materials before and after immersion in BA or PBS for different time intervals was obtained by 3D analysis using CTAn software. The percentage of volumetric change was determined using the following formula: [Percentage of volumetric change = (Repair material volume after immersion *100/Initial volume) -100].^18^ The porosity was quantified as the total porosity percentage.^11^

The percentage of gaps at the material/dentin interface was determined based on the methodology described by a previous study.^20^ The 3D distribution of interface gaps within a predefined volume of interest (VOI) was calculated after filling and after immersion in butyric acid or PBS for 7 and 28 days. For each sample, the evaluation limits (bottom and top) were defined. Adaptive thresholding was applied to recognize each object of interest during binarization. After that, a specific *“task list”* with arithmetic and logical operations between the superimposed sections was used to define the interface volume, including part of the dentin and part of the filling material.^11,21,22^ Within this 3D-VOI, gaps with sizes starting at 8.74 μm were detected.

### Surface analysis by SEM

After 28 days of immersion, three specimens per immersion solution (from n = 5 within each cement group) were selected. Selection was based on post-obturation material volume (mm^3^), specifically the lowest, median, and highest values. This approach was used to reflect the distribution within each solution. Samples were dried with absorbent paper and kept in a desiccator (Plena-Lab, São Paulo, Brazil) containing silica for 30 days. The specimens were coated with carbon and viewed by SEM (JEOL, JSM – 6610LV Scanning Electron Microscope, Peabody, USA) at 12 kV and SS 40. The surface of each sample was examined using three magnifications (x27, x100, and x500).

**Figure 2:**
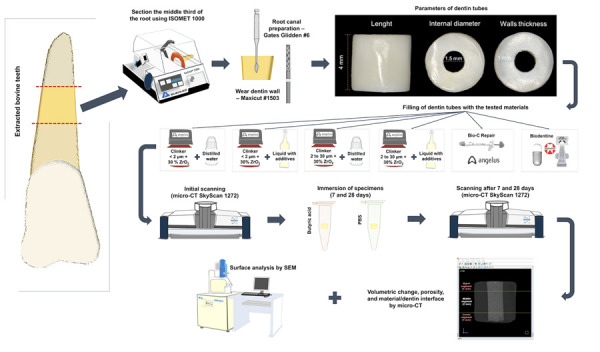
Schematic representation of the methodology used in this study to evaluate the physical properties and surface analysis of various repair materials using micro-CT and SEM.

The methodology used in this study to assess the physical properties and surface analysis of the various repair materials is illustrated in Figure 2.

### Statistical analysis

All data were tested for normality using the Shapiro–Wilk test. Variables related to the percentage of gaps at the material/dentin interface and porosity in the upper and lower segments of the samples after 28 days followed a normal distribution. ANOVA and Tukey tests were used to compare groups, and the unpaired *t*-test was applied to compare immersion solutions. The paired *t*-test was used to compare materials before and after immersion periods.

Variables related to the percentage of volumetric change, porosity after 7 days of immersion, porosity in the middle segment after 28 days, and the percentage increase in porosity after immersion in butyric acid or PBS did not follow a normal distribution. The Kruskal–Wallis with Dunn’s test was applied to compare groups, while the Mann–Whitney test was used to compare immersion solutions. The Wilcoxon test was used to compare materials before and after immersion periods. A level of 5% significance was adopted for all analyses.

## Results

### Volumetric change, porosity, and material/dentin interface

**Figure 3:**
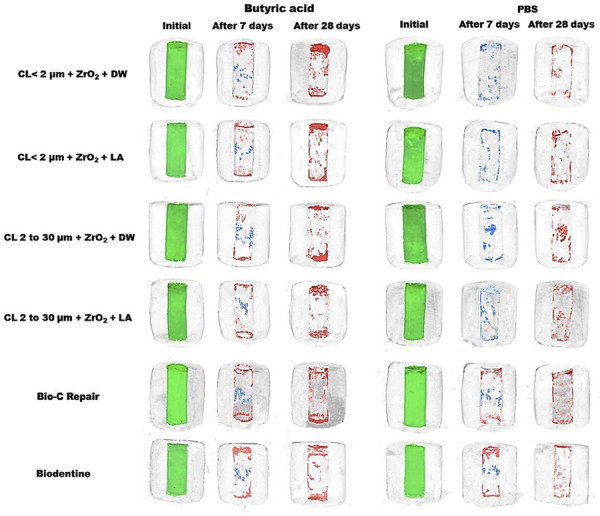
3D models showing the initial volume (green), volume gain (blue), and volumetric loss (red) of the materials evaluated before and after immersion in butyric acid or PBS for a periods of 7 and 28 days.

All cements showed similar volumetric changes to BCR and BIO irrespective of the immersion solutions and evaluation time interval (p > 0.05) (Figure 3, Tables 2 and 3). In the upper and lower segments, CL<2+ZrO_2_+DW showed a greater volumetric loss at 7 and 28 days compared with CL2 to 30+ZrO_2_+LA (p < 0.05) (Figure 3). At 7 days, immersion in butyric acid induced volumetric loss in BCR and CL< 2 µm+ZrO_2_ manipulated with DW or LA (p < 0.05). At 28 days, butyric acid further increased volumetric loss in all groups (p < 0.05) (Table 3).

**Table 2:** Median, minimum, and maximum values of the percentage of volumetric change of materials evaluated after immersion in butyric acid (BA) or PBS for 7 days.

Variables	BA	PBS
Upper and lower segments		
CL < 2 µm + ZrO_2_ + DW	-4.1 (-14.8-(-0.0))^bB^	1.6 (-1.5-3.0)^aA^
CL < 2 µm + ZrO_2_+ LA	-2.5 (-5.7-(-1.1))^abB^	0.1 (-1.4-2.5)^abA^
CL 2 to 30 µm + ZrO_2_+ DW	-1.9 (-6.8-1.1)^abA^	0.2 (-5.4-4.7)^abA^
CL 2 to 30 µm + ZrO_2_+ LA	-1.3 (-3.7-0.4)^aA^	0.6 (-3.5-3.7)^aA^
Bio-C Repair	-4.4 (-5.7-(-0.4))^bB^	-0.1 (-2.2-1.2)^abA^
Biodentine	-3.4 (-8.1-(-0.6))^abA^	-1.1 (-5.8-0.6)^bA^
Middle segment		
CL < 2 µm + ZrO_2_ + DW	3.5 (2.8-3.7)^aA^	0.4 (-1.3-1.2)^bB^
CL < 2 µm + ZrO_2_+ LA	1.8 (1.8-3.7)^aA^	3.5 (1.7-3.7)^aA^
CL 2 to 30 µm + ZrO_2_+ DW	3.0 (1.7-3.7)^aA^	3.5 (3.4-3.6)^aA^
CL 2 to 30 µm + ZrO_2_+ LA	1.8 (1.8-3.8)^aA^	3.6 (1.8-3.6)^aA^
Bio-C Repair	3.5 (1.7-3.6)^aA^	3.6 (1.8-3.7)^aA^
Biodentine	2.7 (1.8-3.4)^aA^	1.8 (1.6-3.8)^aA^

CL: Clinker; ZrO_2_: zirconium oxide; DW: distilled water; LA: liquid with additives; PBS: phosphate-buffered saline. Negative values: volumetric loss. Positive values: volumetric gain. Different superscript lowercase letters in the same column indicate a significant difference between groups (p < 0.05). Different superscript uppercase letters in the same line indicate a significant difference between the immersion solutions (p < 0.05).

**Table 3:** Median, minimum, and maximum values of the percentage of volumetric change of materials evaluated after immersion in butyric acid (BA) or PBS for 28 days.

Variable	BA	PBS
Upper and lower segments		
CL < 2 µm + ZrO_2_ + DW	-20.2 (-41.3-(-8.6))^aB^	-0.1 (-1.7-4.3)^aA^
CL < 2 µm + ZrO_2_+ LA	-15.1 (-5.7-(-1.1))^abB^	-1.3 (-12.5-3.8)^aA^
CL 2 to 30 µm + ZrO_2_+ DW	-12.8 (-34.0-(-4.4))^abB^	-1.0 (-12.5-3.2)^aA^
CL 2 to 30 µm + ZrO_2_+ LA	-12.7 (-19.1-(-5.1))^bB^	-0.9 (-3.5-1.4)^aA^
Bio-C Repair	-13.5 (-32.8-(-9.1))^abB^	-1.1 (-6.9-3.7)^aA^
Biodentine	-15.1 (-37.1-(-9.1))^abB^	-0.1 (14.2-3.1)^aA^
Middle segment		
CL < 2 µm + ZrO_2_ + DW	-0.9 (-2.9-(-0.3))^aA^	-0.7 (-3.2-(-0.3))^aA^
CL < 2 µm + ZrO_2_+ LA	-0.5 (-1.2-0.1)^abA^	-1.0 (-1.9-0.8)^aA^
CL 2 to 30 µm + ZrO_2_+ DW	-0.4 (-3.6-3.3)^abA^	-3.1 (-6.4-(-0.2))^aA^
CL 2 to 30 µm + ZrO_2_+ LA	-0.1 (-0.2-0.8)^bA^	-1.7 (-1.9-(-0.4))^aB^
Bio-C Repair	-0.4 (-0.7-0.4)^abA^	-0.2 (-2.1-0.7)^aA^
Biodentine	-0.3 (-1.1-1.1)^abA^	-0.4 (-8.5-0.1)^aA^

CL: Clinker; ZrO_2_: zirconium oxide; DW: distilled water; LA: liquid with additives; PBS: phosphate-buffered saline. Negative values: volumetric loss. Positive values: volumetric gain.

Different superscript lowercase letters in the same column indicate a significant difference between groups (p < 0.05). Different superscript uppercase letters in the same line indicate a significant difference between the immersion solutions (p < 0.05).

**Figure 4:**
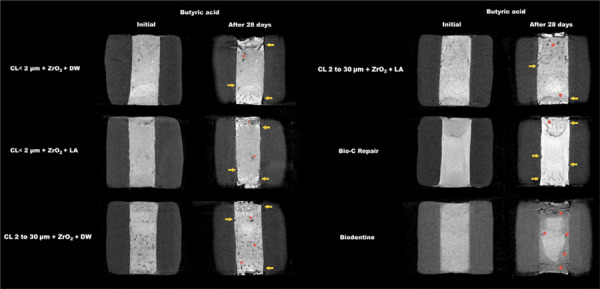
Representative images of porosity (red asterisks) and gaps in the material/dentin interface (yellow arrows) of the different groups after immersion in butyric acid for a time interval of 28 days.

**Figure 5:**
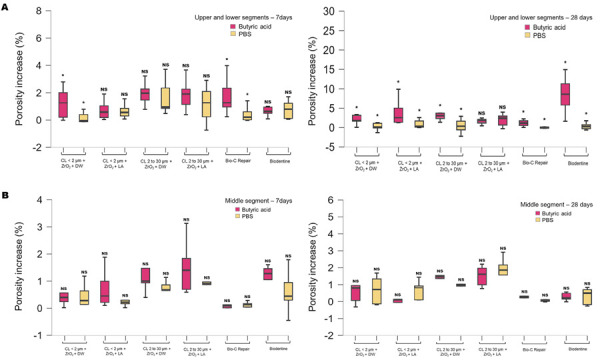
Boxplots showing the median, minimum, and maximum of the porosity increase (%) at the upper and lower segments (A) and middle segment (B) of the samples filled with repair materials after immersion in butyric acid or PBS for time intervals of 7 and 28 days. Asterisk (*) indicates the statistical difference between immersion solutions (p<0.05). NS indicates non-significance between butyric acid and PBS (p>0.05).

All groups showed a higher percentage of porosity and gaps in both evaluation time intervals when compared with baseline (p < 0.05), except for BCR, which showed porosity similar to that of baseline after 28 days (p > 0.05) (Figure 4). In the upper and lower segments at 28 days, porosity under butyric acid increased by approximately 8% throughout all groups, except for CL2 to 30+ZrO_2_+LA, whereas PBS produced only about a 2% increase (p < 0.05) (Figure 5). Under these acidic conditions, the quality at the material/dentin interface was also negatively affected (p < 0.05) (Table 7). Furthermore, CL2 to 30+ZrO_2_+DW and BIO showed higher porosity in butyric acid than PBS within these segments (p < 0.05) (Tables 4 and 5). Whereas in the middle segment, porosity at 28 days was higher than at baseline (p < 0.05), but the percentage increase after butyric acid was similar to that of PBS (p > 0.05) (Table 5, Figure 5).

**Table 7:** Mean and ± standard deviation of the percentage of gaps in the material/dentin interface observed in the end segments (upper and lower) and the middle segment of the materials evaluated before and after immersion in butyric acid (BA) or PBS for 28 days.

Variables	Before immersion		After immersion	
	BA	PBS	BA	PBS
Upper and lower segments				
CL < 2 µm + ZrO_2_ + DW	3.5 ± 0.7^bA^	2.9 ± 0.9^bA^	13.8 ± 4.1^abB*^	7.2 ± 2.8^abB*^
CL < 2 µm + ZrO_2_+ LA	3.2 ± 1.5^bA^	3.4 ± 0.6^bcA^	13.1 ± 3.4^bB*^	7.2 ± 2.9^abB*^
CL 2 to 30 µm + ZrO_2_+ DW	6.2 ± 1.9^aA^	5.5 ± 1.1^aA^	17.5 ± 1.9^aB*^	9.2 ± 1.7^abB*^
CL 2 to 30 µm + ZrO_2_+ LA	4.8 ± 0.8^abA^	4.6 ± 1.8^abA^	14.5 ± 2.7^abB*^	9.8 ± 3.1^abB*^
Bio-C Repair	3.8 ± 0.8^bA*^	2.8 ± 0.8^cA*^	12.5 ± 3.1^bB*^	6.0 ± 1.7^bB*^
Middle segment				
CL < 2 µm + ZrO_2_ + DW	3.2 ± 0.5^bB^	3.9 ± 1.3^aB^	11.5 ± 2.1^aA*^	7.2 ± 3.1^aA*^
CL < 2 µm + ZrO_2_+ LA	3.1 ± 1.7^bB^	3.2 ± 0.9^aB^	6.9 ± 1.9^bA^	7.3 ± 3.4^aA^
CL 2 to 30 µm + ZrO_2_+ DW	6.3 ± 1.6^aB^	5.2 ± 1.8^aB^	10.1 ± 3.2^abA^	9.9 ± 1.8^aA^
CL 2 to 30 µm + ZrO_2_+ LA	4.7 ± 0.7^abB^	4.5 ± 2.1^aB^	8.4 ± 1.9^abA^	9.1 ± 3.3^aA^
Bio-C Repair	3.8 ± 0.8^bB^	2.7 ± 0.9^aB^	8.4 ± 1.1^abA*^	5.7 ± 1.5^aA*^

CL: Clinker; ZrO_2_: zirconium oxide; DW: distilled water; LA: liquid with additives; PBS: phosphate-buffered saline. Different superscript lowercase letters in the same column indicate a significant difference between groups (p < 0.05). Different superscript uppercase letters in the same line indicate a significant difference between before and after immersion (p < 0.05). * Indicates a significant difference between immersion solutions (p < 0.05).

**Table 4:** Median, minimum, and maximum values of the percentage of porosity observed in the end segments (upper and lower) and the middle segment of the materials evaluated before and after immersion in butyric acid (BA) or PBS for 7 days.

Variables	Before immersion		After immersion	
	BA	PBS	BA	PBS
Upper and lower segments				
CL < 2 µm + ZrO_2_ + DW	0.8 (0.1-1.8)^bB^	1.5 (0.2-3.7)^bcB^	1.8 (0.2-8.6)^bcA^	2.0 (0.1-3.8)^bA^
CL < 2 µm + ZrO_2_+ LA	0.6 (0.1-4.4)^bB^	0.8 (1.1-3.0)^bcB^	1.4 (1.2-6.3)^bcA^	1.4 (0.2-4.5)^bA^
CL 2 to 30 µm + ZrO_2_+ DW	4.1 (2.5-11.1)^aB^	5.3 (1.1-9.7)^aB^	6.5 (4.2-13.1)^aA^	7.0 (2.9-11.7)^aA^
CL 2 to 30 µm + ZrO_2_+ LA	2.0 (0.8-4.7)^aB^	1.1 (1.3-4.9)^bB^	4.5 (1.6-5.8)^abA^	3.4 (1.5-7.8)^aA^
Bio-C Repair	0.6 (0.0-2.1)^bB^	0.8 (0.0-4.3)^bcB^	2.3 (0.2-5.1)^bA^	1.1 (0.1-5.0)^bA^
Biodentine	0.5 (0.1-1.5)^bB^	0.9 (0.3-3.8)^cB^	1.2 (0.5-4.5)^bcA^	1.8 (0.4-4.6)^bA^
Middle segment				
CL < 2 µm + ZrO_2_ + DW	0.3 (0.1-1.6)^bc^	0.9 (0.3-1.6)^bc^	0.6 (0.1-4.5)^bc^	0.9 (0.6-2.4)^bc^
CL < 2 µm + ZrO_2_+ LA	0.1 (0.0-1.1)^c^	0.1 (0.0-1.1)^c^	0.2 (0.1-2.0)^c^	0.2 (0.0-2.3)^c^
CL 2 to 30 µm + ZrO_2_+ DW	4.2 (2.6-4.7)^a^	3.5 (2.4-6.9)^a^	5.7 (3.1-7.7)^a^	4.4 (3.0-7.1)^a^
CL 2 to 30 µm + ZrO_2_+ LA	1.9 (0.7-2.7)^ab^	2.1 (1.4-3.5)^ab^	2.6 (2.3-5.2)^ab^	2.5 (2.2-6.4)^ab^
Bio-C Repair	0.1 (0.0-1.5)^c^	0.6 (0.0-0.9)^c^	0.2 (0.0-2.1)^c^	0.8 (0.0-1.2)^c^
Biodentine	0.1 (0.1-1.4)^bc^	0.6 (0.1-1.7)^bc^	1.6 (0.4-2.9)^bc^	1.2 (0.8-2.4)^bc^

CL: Clinker; ZrO_2_: zirconium oxide; DW: distilled water; LA: liquid with additives; PBS: phosphate-buffered saline. Different superscript lowercase letters in the same column indicate a significant difference between groups (p < 0.05). Different superscript uppercase letters in the same line indicate a significant difference between before and after immersion in the upper and lower segments (p < 0.05). There was no significant difference between the immersion solutions in the upper and lower segments of the samples (p > 0.05). There was no significant difference between the immersion solutions and between before and after immersion in the middle segment of the samples (p > 0.05).

**Table 5:** Percentage of porosity of the materials evaluated before and after immersion in butyric acid (BA) or PBS for 28 days.

Variable	Before immersion		After immersion	
	BA	PBS	BA	PBS
Upper and lower segments				
CL < 2 µm + ZrO_2_ + DW	1.0 ± 0.5^bA*^	1.3 ± 0.6^bA^	2.4 ± 1.1^cA*^	1.7 ± 0.8^cA^
CL < 2 µm + ZrO_2_+ LA	1.1± 0.8^bA*^	1.6 ± 1.1^bA^	4.2 ± 2.3^bcA*^	2.5 ± 1.1^bcA^
CL 2 to 30 µm + ZrO_2_+ DW	5.9 ± 2.4^aA*^	5.4 ± 2.1^aA^	9.7 ± 4.2^aA*^	6.1 ± 2.1^aB^
CL 2 to 30 µm + ZrO_2_+ LA	2.1 ± 1.2^bA*^	3.1 ± 1.1^bA*^	3.7 ± 1.1^cA*^	5.0 ± 2.1^aA*^
Bio-C Repair	1.0± 0.5^bA^	1.5 ± 0.5^bA^	1.8 ± 1.0^cA^	1.6 ± 1.1^cA^
Biodentine	0.4± 0.1^bA*^	1.1 ± 0.9^bA^	8.8 ± 3.5^abA*^	1.2 ± 0.4^cB^
Middle segment				
CL < 2 µm + ZrO_2_ + DW	1.0 (0.2-1.5)^bA^	0.7 (0.2-1.2)^bA^	2.3 (0.3-6.2)^abA^	0.9 (0.6-2.6)^bcA^
CL < 2 µm + ZrO_2_+ LA	0.1 (0.1-1.0)^bcB^	1.6 (0.3-3.1)^abA^	0.2 (0.1-0.7)^cB^	2.5 (0.4-4.6)^abA^
CL 2 to 30 µm + ZrO_2_+ DW	4.2 (2.6-4.6)^abA*^	2.9 (2.4-5.9)^aA^	5.7 (4.1-6.5)^aA*^	5.6 (1.0-6.5)^aA^
CL 2 to 30 µm + ZrO_2_+ LA	1.4 (0.6-2.2)^bB^	3.1 (2.2-4.1)^aA*^	2.8 (1.7-4.4)^abB^	4.9 (3.1-7.9)^aA*^
Bio-C Repair	0.3 (0.0-1.1)^bcA^	0.6 (0.1-0.9)^bA^	1.3 (0.0-1.6)^bcA^	0.6 (0.0-1.0)^cA^
Biodentine	0.1 (0.0-0.3)^cA*^	0.3 (0.1-1.7)^bA^	0.5 (0.1-0.6)^cA*^	0.8 (0.1-1.5)^cA^

Mean and ± standard deviation of the percentage of porosity in the upper and lower segments. Median, minimum, and maximum values of the percentage of porosity in the middle segment. CL: Clinker; ZrO_2_: zirconium oxide; DW: distilled water; LA: liquid with additives; PBS: phosphate-buffered saline. Different superscript lowercase letters in the same column indicate a significant difference between groups (p < 0.05). Different superscript uppercase letters in the same line indicate a significant difference between the immersion solutions (p < 0.05). * Indicates the difference between before and after immersion (p < 0.05).

Within the experimental groups, CL2 to 30+ZrO_2_+DW demonstrated higher porosity values compared with the other groups in all evaluation time intervals (p < 0.05) (Figure 4), and a higher percentage of gaps at the interface compared with the other groups at the upper and lower segments of the samples (p < 0.05). (Tables 6 and 7). Overall, immersion in butyric acid for 28 days led to greater volumetric loss, increased porosity, and higher number of interfacial gaps than those in PBS, especially in the upper and lower segments.

**Table 6:** Mean and ± standard deviation of the percentage of gaps in the material/dentin interface observed in the end segments (upper and lower) and the middle segment of the materials evaluated before and after immersion in butyric acid (BA) or PBS for 7 days.

Variables	Before immersion		After immersion	
	BA	PBS	BA	PBS
Upper and lower segments				
CL < 2 µm + ZrO_2_ + DW	3.5 ± 0.7^bA^	2.9 ± 0.9^bcA^	6.0 ± 1.3^bcB^	5.1 ± 1.5^abB^
CL < 2 µm + ZrO_2_+ LA	3.2 ± 1.5^bA^	3.4 ± 0.6^bcA^	5.7 ± 1.2^cB^	4.7 ± 1.1^bB^
CL 2 to 30 µm + ZrO_2_+ DW	6.2 ± 1.9^aA^	5.5 ± 1.1^aA^	9.2 ± 1.5^aB^	7.1 ± 1.8^aB^
CL 2 to 30 µm + ZrO_2_+ LA	4.8 ± 0.8^abA^	4.6 ± 1.8^abA^	8.1 ± 1.4^abB^	6.1 ± 1.8^abB^
Bio-C Repair	3.8 ± 0.8^bA*^	2.8 ± 0.8^cA*^	5.8 ± 1.1^bcB*^	3.8 ± 1.1^bB*^
Middle segment				
CL < 2 µm + ZrO_2_ + DW	3.2 ± 0.5^bB^	3.9 ± 1.3^aB^	5.6 ± 1.3^abA^	4.9 ± 2.3^aA^
CL < 2 µm + ZrO_2_+ LA	3.1 ± 1.7^bB^	3.2 ± 0.9^aB^	5.1 ± 1.9^bA^	4.7 ± 1.3^aA^
CL 2 to 30 µm + ZrO_2_+ DW	6.3 ± 1.6^aB^	5.2 ± 1.8^aB^	8.4 ± 2.4^aA^	7.0 ± 1.8^aA^
CL 2 to 30 µm + ZrO_2_+ LA	4.7 ± 0.7^abB^	4.5 ± 2.1^aB^	7.9 ± 1.4^abA^	5.9 ± 2.6^aA^
Bio-C Repair	3.8 ± 0.8^bB^	2.7 ± 0.9^aB^	5.7 ± 1.3^abA*^	3.6 ± 1.3^aA*^

CL: Clinker; ZrO_2_: zirconium oxide; DW: distilled water; LA: liquid with additives; PBS: phosphate-buffered saline. Different superscript lowercase letters in the same column indicate a significant difference between groups (p < 0.05). Different superscript uppercase letters in the same line indicate a significant difference between before and after immersion (p < 0.05). * Indicates a significant difference between immersion solutions (p < 0.05).

### Surface analysis by SEM

**Figure 6:**
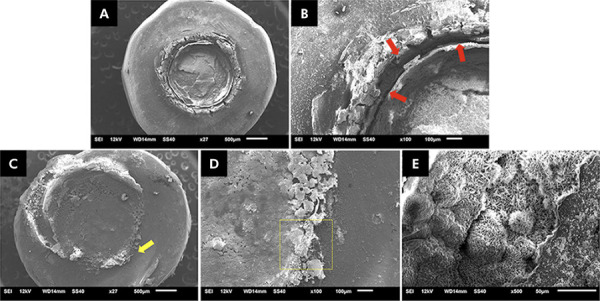
SEM images. Dentin tube filled with calcium silicate-based repair material after immersion in butyric acid for 28 days – x27 magnification (A) and formation of cracks in the material/dentin interface (red arrows) – x100 magnification (B). Dentin tube filled with calcium silicate-based repair material after immersion in PBS (yellow arrow) – x27 magnification (C); higher magnification (yellow square) demonstrates adaptation in the material/dentin interface - x100 magnification (D), and higher magnification demonstrates the formation of hydroxyapatite crystals on the surface in the interface - x500 magnification (E).

Descriptive analysis by SEM revealed differences in surface morphology and the material/dentin interface according to the type of immersion (Figure 6). In PBS, the material surface image at x500 clearly exhibited the formation of hydroxyapatite crystals. In addition, a continuous interfacial layer between the material and dentin was observed. Whereas immersion in butyric acid at magnification x100 demonstrated structural loss, material corrosion, and cracks at the material/dentin interface. These findings were consistently observed throughout all cements, suggesting the bioactive potential of these materials in PBS, and their harmful impact under acidic conditions.

## Discussion

Calcium silicate–based materials generally maintain bioactivity and surface stability under neutral pH conditions.^19^ However, in infection and inflammation, acidic variations can alter their physicochemical properties and compromise long-term performance.^13,15^ Previous studies have investigated these effects mainly in other repair cements,^13,15-19^ but none have specifically evaluated Clinker Angelus under acidic challenge. The present study, in which a standardized dentin-tube model was used, is the first to assess the physical properties of Clinker after immersion in butyric acid or PBS. Our results demonstrated that acidic pH significantly impaired Clinker stability, particularly when manipulated with distilled water, underscoring the importance of appropriate material selection under clinical conditions involving an acidic environment.

Immersion in butyric acid caused greater volumetric loss, porosity, and interface gaps in all materials compared with PBS, leading to the rejection of the first null hypothesis. The pH value of a periapical lesion depends on the host´s inflammatory response and bacterial byproducts.^12^ However, the presence of an acidic environment impairs hydration and the formation of carbonated hydroxyapatite in calcium silicate cements,^13^ which explains the structural degradation observed in our study. A previous investigation demonstrated that butyric acid (pH 4.4) significantly reduced the surface microhardness of White ProRoot MTA.^15^ Based on these findings, butyric acid (pH 4.1) was selected as a clinically relevant model. It is one of the metabolic byproducts of anaerobic bacteria involved in inflammatory processes.^23^ This compound mimics the acidic conditions of endodontic infections.

Our results revealed that all experimental groups had similar volumetric stability to that of BCR and BIO irrespective of the immersion solutions and evaluation time interval. Long-term dimensional stability is essential for maintaining the sealing ability of repair materials.^2^ Consistent with previous reports, BCR and BIO showed volumetric stability when stored in distilled water or PBS,^3,19,24,25^ but both exhibited volumetric loss under acidic conditions.^13,17,19^ In contrast, the present study is the first to report how Clinker Angelus responds volumetrically to an acidic challenge, expanding current knowledge on calcium silicate–based repair materials.

The second null hypothesis was rejected since CL<2+ZrO_2_+DW exhibited greater volumetric loss than CL2 to 30+ZrO_2_+LA in the upper and lower segments at both 7 and 28 days (Figure 3). This behavior may be explained by particle size and the type of liquid used. Smaller particles promote higher contraction during hydration,^26^ compromising sealing ability, and they dissolve more rapidly in water, accelerating the setting reaction.^8^ In addition, BCR and CL<2 groups manipulated with DW or LA also showed volumetric loss after 7 days. The presence of plasticizer agents in the liquid of hydraulic materials has been associated with mass loss,^10^and their surfactant effect disperses cement particles,^27^ which may account for the volumetric instability observed. Unlike previous studies that investigated particle size and liquid effects through solubility tests, the present study demonstrated these relationships using micro-CT analysis, providing novel insights into the volumetric stability of Clinker Angelus.

Low porosity and adequate sealing at the material/dentin interface are critical to minimizing bacterial infiltration.^11^ In this study, CL2 to 30+ZrO_2_+DW exhibited greater porosity and larger gaps at the material/dentin interface compared with the other groups (Figure 4). This can be explained by the incomplete hydration of larger cement particles, which leads to a greater presence of voids due to the limited participation of hydration products.^28^ Whereas BIO presented high porosity throughout all segments (Figure 4), in contrast with previous studies that reported lower values when it was immersed in PBS or butyric acid.^13,24^ This difference may be related to the powder-liquid ratio used in our study (1 capsule: 6 drops of liquid), which was slightly higher than it was in those reports (1 capsule: 5 drops of liquid),^13,24^ since increased liquid content has been associated with greater porosity.^28^ BCR showed lower porosity but a higher percentage of interfacial gaps after immersion in butyric acid. These results differed from the *in vivo* findings, in which minimal gaps were observed after subcutaneous implantation.^11^ Such divergences may be explained by methodological differences, including the type of fluid, pH conditions, and contact of samples with the materials, which influence porosity and interfacial gap formation.

Longer immersion periods allow a better understanding of the behavior of hydraulic materials, as hydration continues even after the final setting time.^29^ The 7- and 28-day time intervals were selected to reflect the initial and long-term material characteristics, consistent with previous investigations on calcium silicate–based cements.^11,19,25^ Our results showed that immersion in butyric acid for 28 days influenced volumetric change, porosity, and the material/dentin interface compared with 7 days. These findings align with previous reports of volume loss and increased interface gaps after 30 and 60 days, respectively.^11,19,25^ Therefore, the third null hypothesis was rejected. Importantly, this is the first study to evaluate the effects of butyric acid and PBS on these properties in calcium silicate–based repair cements after 28 days, which may serve as a basis for future investigations.

SEM is a recognized method for assessing calcium silicate-based materials, providing detailed information on external morphology, chemical composition, and crystal structure.^4,30,31^ In this investigation, SEM analysis revealed structural loss, corrosion, and cracks at the material/dentin interface when exposed to butyric acid. This finding reinforces the premise that an acidic pH interferes with material hydration and reduces apatite deposition on the surface and at the material/dentin interface.^13^Similarly, a previous study reported increased bubbles and surface irregularities in repair materials after acid exposure.^19^ Whereas formation of hydroxyapatite crystals on the material surface and tag formation at the material/dentin interface were observed when immersed in PBS (Figure 6), in agreement with previous studies,^4,30^ supporting the biomineralization potential of these materials. SEM and micro-CT provided complementary insights. Micro-CT was used to quantify volumetric stability, porosity, and interfacial gaps in three dimensions. SEM revealed the underlying morphological and structural changes. Taken together, these powerful approaches offered a comprehensive understanding of how different immersion solutions affect the physical stability of calcium silicate–based repair cements.

In terms of methodology, standardized dentin tube models prepared from bovine teeth were used to evaluate the interaction of calcium silicate materials with dentin walls. Although bovine and human dentin present structural and compositional differences, the use of bovine substrates in push-out, fracture-resistance, microleakage, and shear bond-strength assays has been widely validated, supporting their translational relevance.^31,32^ Nevertheless, extrapolation of these findings to human dentin should be made with caution.^31,32^ Analyses of different segments of the specimen (upper, middle, and lower segments – Figure 1B) allowed monitoring of the behavior of materials inside the mass and those close to the tissue fluid.^11^ Additionally, the evaluation of material properties at different time intervals was carried out using high-resolution micro-CT, facilitating measurement of the volume and difference between the filling material, voids, and tooth structure.^33^ Although micro-CT is a high-precision technique and is widely regarded as a reference standard for assessing the physical and morphological properties of endodontic materials,^2,3,11,13,17^ image quality is influenced by multiple factors, one of which is the isotropic voxel size.^34^ Consequently, interfacial defects smaller than 8.74 μm, the voxel size used in this study, may not be detected.

Despite its contributions, this study has limitations that should be acknowledged. The descriptive SEM analysis was based on a limited number of specimens. Such a small sample size may not capture the full variability of material behavior, reinforcing the need for cautious interpretation and validation with larger datasets.^35^ As regards the materials, BIO was included as a comparison due to its adequate physicochemical properties.^25^ However, the analysis at the material/dentin interface was not possible. Its low radiopacity (2.30 mm Al),^25^ which is similar to that of root dentin (2.27 mm Al),^36^ impaired the segmentation process in micro-CT images.^31^ Future studies using alternative methodologies, such as confocal microscopy or leakage assays, may provide valuable insights. The immersion environment adopted in this study was selected since it simulates clinical conditions, as reported in several studies.^2,13,15^ Nevertheless, in actual inflammatory scenarios, pH levels fluctuate over time and the duration of acidic exposure remains uncertain,^37^ representing a challenge to reproducing dynamic biological conditions in vitro. Finally, it is important to emphasize that this was an in vitro investigation, providing only a preclinical perspective on the repair materials. Future in vivo and in vitro studies are needed to evaluate additional physicochemical and biological properties of Clinker Angelus under different pH conditions.

## Conclusions

Acidic pH significantly increased porosity, dimensional change, and material/dentin interface gaps in calcium silicate–based repair cements. Clinker Angelus, manipulated with distilled water, exhibited the highest porosity and gap percentages, as well as the greatest volume loss, indicating compromised structural stability. These findings highlight the importance of both the manipulation protocol and the clinical environment in determining the long-term performance of calcium silicate–based materials.

## Data Availability

The datasets generated during and/or analyzed during the current study are available from the corresponding author on reasonable request.
